# Initial Diagnostic Strategies for Helicobacter Pylori in Patients With Bleeding Peptic Ulcers Undergoing Endoscopy: A Cost-Effectiveness Analysis

**DOI:** 10.1016/j.gastha.2024.100602

**Published:** 2024-12-15

**Authors:** Michael G. Artin, Josephine Soddano, Sheila D. Rustgi, Zainab Aziz, Francesca Lim, Jeong Yun Yang, Myles A. Ingram, John T. Nathanson, John Y. Kao, Chin Hur

**Affiliations:** 1 Department of Medicine, Hospital of the University of Pennsylvania, Philadelphia, Pennsylvania; 2 Department of Medicine, Columbia University Irving Medical Center, New York, New York; 3 Division of Digestive and Liver Diseases, Department of Medicine, Columbia University Irving Medical Center, New York, New York; 4 Division of Gastroenterology, Department of Internal Medicine, University of Michigan Medical School, Ann Arbor, Michigan

**Keywords:** H. pylori, Peptic Ulcer Disease, GI Bleeding, Hospitalization, Cost-Effectiveness Analysis

## Abstract

**Background and Aims:**

Helicobacter pylori (H. pylori) is a major cause of peptic ulcer disease (PUD) and upper gastrointestinal bleeding. Testing for and eradication of H. pylori reduces the risk of future PUD-related complications including readmission for gastrointestinal bleeding. Our aim was to determine the most cost-effective testing strategy for H. pylori in patients hospitalized with bleeding peptic ulcers.

**Methods:**

We developed a Markov cohort model to compare the following 6 H. pylori testing strategies: no testing, histology, rapid urease test, stool antigen test, urea breath test (UBT), and serology. Histology and rapid urease test require biopsies, while stool antigen test, UBT, and serology do not. We assumed a 17% H. pylori prevalence in patients admitted with bleeding ulcers. Model outcomes included hospitalizations for rebleeds, number needed to treat to avoid another hospitalization, life expectancy, total cost, quality-adjusted life years, and incremental cost-effectiveness ratios.

**Results:**

Compared to no testing, UBT resulted in a gain of 0.02 quality-adjusted life years, total cost savings of $2140 per patient, and 1675 hospitalizations avoided per 10,000 patients per year. Additionally, the number needed to treat to avoid an additional hospitalization over 35 years was 167. UBT was the preferred strategy as it was both less costly and more effective than no testing.

**Conclusion:**

Our findings suggest that UBT is the cost-effective strategy to identify H. pylori in patients admitted with PUD. Noninvasive H. pylori testing at the point of care or during inpatient admission should be considered, as it presents limited risk to patients and offers potential clinical benefits.

## Introduction

Peptic ulcer disease (PUD), characterized by ulcers of the gastrointestinal (GI) tract, is a significant source of morbidity and mortality worldwide.^1^ Helicobacter pylori (H. pylori) is a leading cause of PUD and upper GI bleeding. Testing and eradication of H. pylori are critical interventions to reduce the risk of future PUD-related complications including rebleeding and hospital readmissions. However, testing in the acute inpatient setting has been hindered for various reasons, including the theoretical risk of lowered sensitivity of H. pylori tests with concomitant proton pump inhibitor (PPI) therapy and the concern that gastric biopsies could increase the risk of additional GI bleeding.^2^, ^3^, ^4^

It is estimated that over 50% of the global population is affected by H. pylori, although its prevalence varies widely by region.^5^ The Centers for Disease Prevention and Control estimate that H. pylori prevalence in developing countries is approximately 70%, while prevalence in industrialized countries like the US ranges between 30% and 40%.^6^ Patients infected with H. pylori commonly present with epigastric pain, anemia, and GI bleeding from ulceration of the stomach and small intestine. However, some individuals may remain asymptomatic.^7^^,^^8^ The most common causes of peptic ulcer formation include H. pylori infection and excessive nonsteroidal anti-inflammatory drug (NSAID) use.^9^^,^^10^ Smoking and excessive alcohol use can also increase the risk of ulceration and bleeding from ulceration.^11^, ^12^, ^13^, ^14^ In the absence of patient history of NSAID use or H. pylori infection status, clinicians cannot reliably differentiate the etiology of ulcers based on gross appearance endoscopically without further diagnostic testing.^15^, ^16^, ^17^, ^18^

H. pylori can be tested invasively (ie, rapid urease test [RUT] and [histology]) or noninvasively (ie, stool antigen test [SAT], urea breath test [UBT], and serology). However, the optimal test in the context of a bleeding peptic ulcer is unknown. Invasive tests require tissue biopsy to assess for the presence of urease or H. pylori in histology. RUT detects the presence of ammonia due to a breakdown of urea by the enzyme urease found in H. pylori.^19^ It can be done in as quickly as 1 hour, though at the detriment of test specificity and sensitivity. Noninvasive tests, such as the UBT and the SAT do not require endoscopy and tissue biopsy, while providing alternate, high-fidelity methods for diagnosing H. pylori.^20^ UBT tests for liberated carbon dioxide in the breath via hydrolysis of urea by H. pylori; SAT detects the presence of H. pylori bacterial antigen in the stool and is therefore useful for both diagnosing H. pylori and confirming eradication.^21^^,^^22^ However, compared to the other noninvasive strategies, H. pylori-specific immunoglobulin G serology tests exposure to the bacteria at any point, not necessarily active infection.

In patients who present with symptoms of H. pylori without bleeding, it is recommended that PPIs be stopped 1–2 weeks before testing and antibiotics or bismuth containing medications be stopped 4 weeks before testing to maximize sensitivities of tests for H. pylori.^23^^,^^24^ However, patients presenting with upper GI bleeding are typically given PPIs before H. pylori testing. Furthermore, the sensitivity and specificity of all the testing modalities listed above are decreased by active GI bleeding.^25^ As a result, testing and eradication for H. pylori are typically not achieved at point of care, and subsequent outpatient testing may frequently not occur.^26^^,^^27^

Therefore, it is critical to determine the most appropriate H. pylori test in the setting of peptic ulcer bleeding, to reduce PUD-related complications. In our study, we performed a cost-effectiveness analysis to determine the optimal testing mechanism for H. pylori in the setting of bleeding peptic ulcers in the hospital environment.

## Materials and Methods

### Model Overview

A Markov cohort model was constructed using transition probabilities drawn from literature (Figure 1). Patients entered the model at age 65 hospitalized with an assumed first-time peptic ulcer bleed while undergoing endoscopy. Patients then received 1 of 6 strategies to determine their H. pylori status: no testing, histology, RUT, SAT, UBT, or serology (Immunoglobulin G antibody testing). Patients cycled annually until age 100 or until death, transitioning between possible states of initial infection, eradication, reinfection, and rebleeding. Death was determined by either 30-day mortality from a bleeding peptic ulcer or by all other causes.^28^^,^^29^ Although serology does not indicate active infection, the model assumed that this was the first time a patient was identified as having H. pylori and thus the first time being treated.

**Figure:**
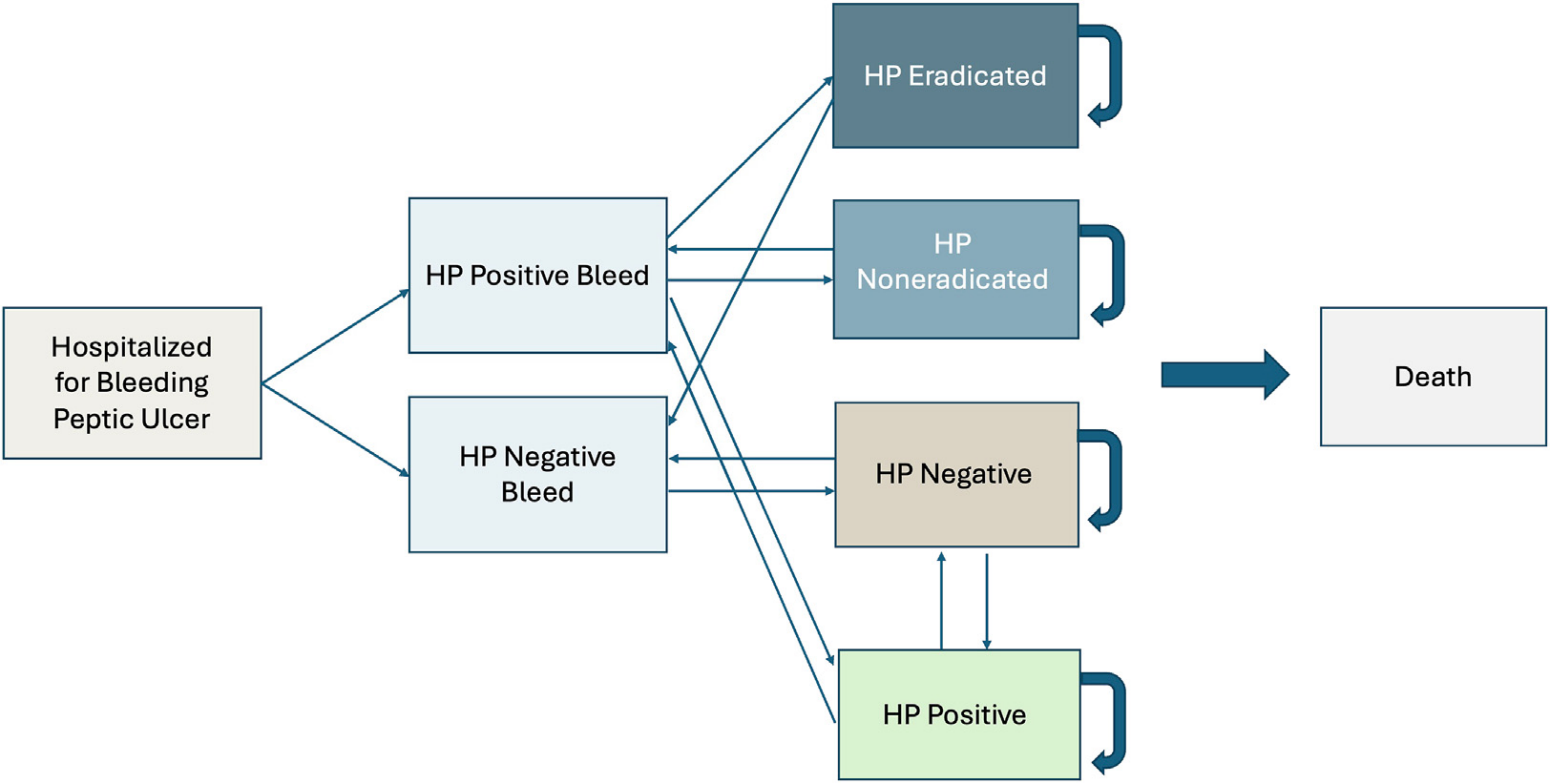
Markov Model schematic. All patients enter the Markov Model at age 65 hospitalized with an assumed first-time peptic ulcer bleed undergoing endoscopy and are tracked annually until age 100 or death. Patients receive 1 of 6 strategies, which may result in a true/false positive/negative: no testing, histology, RUT, SAT, UBT, or serology. Over the course of the model, patients can potentially become reinfected with H. pylori or experience another rebleed.

Patients received Clarithromycin triple antibiotic H. pylori eradication therapy with antibiotics and a PPI after testing positive for H. pylori. If this was unsuccessful, then the patient received quadruple antibiotic eradication therapy. If quadruple antibiotic eradication therapy was also unsuccessful, then the patient transitioned into an H. pylori noneradicated state. For patients who tested negative for H. pylori, a repeat test was not included in the model. Diagnosis and treatment for H. pylori outside of hospitalization for bleeding episodes was not considered. Without strong evidence of the risk of rebleeding for all-cause H. pylori negative ulcers, the risk of rebleeding for H. pylori negative patients in this model was assumed to be that of H. pylori eradicated patients. Due to the late start age of patients in the model and the uncertainty of the benefit for reducing gastric cancer risk after H. pylori eradication, the downstream impact of H. pylori on the future development of gastric cancer was not included.

Currently, there is a lack of literature that reports the prevalence of H. pylori–positive ulcers in inpatient settings. However, recent literature suggests that the prevalence of H. pylori–positive ulcers in outpatient endoscopy clinics is 17%.^30^ Assuming that the prevalence of H. pylori–positive ulcers is similar in both outpatient and inpatient settings, we used 17% H. pylori prevalence in the base case of our model. We also varied H. pylori prevalence levels between 10% and 90% in sensitivity analyses.

### H. pylori Eradication Therapies

Our H. pylori eradication therapy parameters were adopted from the Toronto Guidelines for H. pylori treatment.^31^ Clarithromycin triple antibiotic H. pylori eradication therapy consists of PPI administered twice daily, Clarithromycin (500 mg) administered twice daily, and Amoxicillin (1 gram) administered twice daily. Treatment duration is 14 days.^23^^,^^31^, ^32^, ^33^ Quadruple antibiotic H. pylori eradication therapy consists of PPI administered twice daily, Bismuth subcitrate (120–300 mg or 420 mg) or Bismuth subsalicylate (300 or 524 mg) administered 4 times daily, Tetracycline (500 mg) administered 4 times daily, and Metronidazole (500 mg) administered 3–4 times daily. Quadruple antibiotic eradication therapy duration is 14 days.^23^^,^^31^^,^^32^^,^^34^

### Test Performance and Costs

Base case estimates were obtained from a systematic review and meta-analysis for H. pylori testing performances in the context of bleeding peptic ulcers in the inpatient setting.^35^ We assume that this study reflects the reduced sensitivities of test strategies in the context of PPI use, as most patients are prescribed PPIs at the time of hospitalization. Estimates for the probability, utility, and cost values in our model were obtained from previously published literature (Table A1). Costs of the H. pylori testing strategies were obtained from the Centers for Medicare & Medicaid Services (Table A1).

The endpoints for our analysis were total quality-adjusted life years (QALYs), costs, bleeds avoided, number needed to treat (NNT) to avoid another bleed, and incremental cost-effectiveness ratio (ICER). Total costs comprised of H. pylori testing, H. pylori drug eradication therapy, and hospitalization. ICER is a measurement of additional cost required for gain in QALYs. All costs were adjusted to 2022 US dollars using the Consumer Price Index for health-care costs.^36^ Costs and QALYs were discounted at an annual rate of 3%. We used the commonly accepted US willingness-to-pay (WTP) threshold of $100,000/QALY to determine cost-effectiveness.^37^

### Statistical Analyses

All data processing and statistical analysis were performed with the R statistical computing software (version 4.2.3; R Core Team 2023). To determine the impact of model input uncertainty on cost-effectiveness results, we performed one-way and probabilistic sensitivity analyses. We also considered various scenarios where the H. pylori positive prevalence of bleeding peptic ulcers ranged between 10% and 90%.

In 1-way sensitivity analyses, model parameters were varied one at a time around the base case value, holding all other parameters constant. Upper and lower bounds for parameters were determined from 95% confidence intervals around the parameter mean. All cost ranges in the model came from reported literature, and in the absence of previously reported data upper and lower bounds were set to 20% above and 20% below the mean cost.

In the probabilistic sensitivity analysis, each model input was sampled simultaneously from a probability distribution. The mean values for these distributions were base case values, and standard deviations were determined from literature or the corresponding database. Gamma distributions were used for costs and beta distributions for all other variables. The Gamma distribution is chosen for costs due to its ability to handle non-negative values and capture the skewness often seen in health-care costs, particularly in the context of H. pylori testing strategies. The Beta distribution is appropriate for parameters constrained between 0 and 1 such as diagnostic probabilities. Probabilistic sensitivity analyses were performed over 10,000 iterations for each strategy.

We did not adjust for multiple comparisons, as is typical in cost-effectiveness analyses. Our study focuses on comparing the relative effectiveness of various strategies, rather than evaluating specific hypotheses about their equivalence or superiority.

## Results

### Base Case Results

For our base-case, all noninvasive strategies (SAT, UBT, and serology) were less costly and more effective than the invasive strategies (Histology and RUT). The no testing strategy resulted in 16.58 QALYs and a total cost of $3142. UBT was the cost-effective strategy with cost savings of $2140 and a gain of 0.32 QALYs compared to no testing. The ICER for UBT was −$6786 per QALY gained, with a negative ICER indicating that the strategy was less costly and more effective with respect to the no testing strategy (Table 1). UBT also resulted in 1675 bleeds avoided and a NNT of 5.98 with respect to the referent strategy.

**Table:** ICERs for all 6 Strategies at 17% H. pylori Positive Prevalence in Bleeding Peptic Ulcers

Strategy	Bleeds avoided	NNT	Costs (Testing + drug therapy + hospitalization)	QALYs	ICER
No testing	Ref.	Ref.	$3142	16.581	Ref.
RUT	1316	7.60	$1474	16.818	Dominated
Histology	1364	7.33	$1412	16.828	Dominated
SAT	1602	6.24	$1068	16.880	Dominated
Serology	1615	6.19	$1053	16.883	Dominated
UBT	1675	5.98	$1002	16.896	−6786.27

This table shows the number of bleeds avoided and NNT for each testing strategy with respect to the no testing strategy, costs, QALYs, and ICERs. The no testing strategy was compared against 5 testing strategies: RUT, Histology, SAT, Serology, and UBT.

### Scenario Analyses

We varied the prevalence of H. pylori–positive bleeding peptic ulcers from 10% to 90% (Table A2). Despite changing the prevalence values, UBT remained the cost-effective strategy. Compared to no testing, UBT resulted in incremental QALYs of 0.19–1.31 and total cost savings of $1253.39–11,387.52 per patient, ICERs of −$6596.79/QALY to −$8692.76/QALY, 985–8865 bleeds avoided per 10,000 patients per year, and an NNT of 1.13–10.15 to avoid an additional bleed over 35 years per patient. As H. pylori prevalence increased, the magnitude of the negative ICER also increased. This was due to the increase of QALYs as H. pylori prevalence increased.

Since UBT and serology testing are not always available, we considered a scenario in our model where UBT and serology were not possible testing strategies. In this scenario, SAT became the cost-effective test. Compared to no testing, SAT resulted in an increase of 0.2 QALYs and a cost savings of $2074, with an ICER of −$10370/QALY. Therefore, whenever UBT is not available as a testing option, SAT is the preferred alternative.

### One-Way Sensitivity Analyses

**Figure A1:**
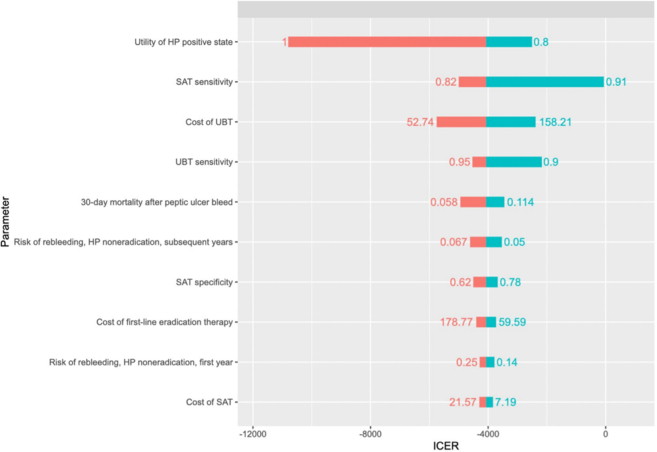
One-way Sensitivity Analysis of UBT vs SAT at 17% H. pylori Prevalence in Bleeding Peptic Ulcers.

We performed a 1-way sensitivity analysis between SAT and UBT since they are the 2 most effective strategies in our model. At an H. pylori prevalence of 17%, UBT remained cost-effective compared to SAT across all ranges of input parameters. The model was most sensitive to the following parameters: utility of H. pylori positive state, SAT sensitivity, cost of UBT, and UBT sensitivity (Figure A1).

### Probabilistic Sensitivity Analyses

**Figure A2:**
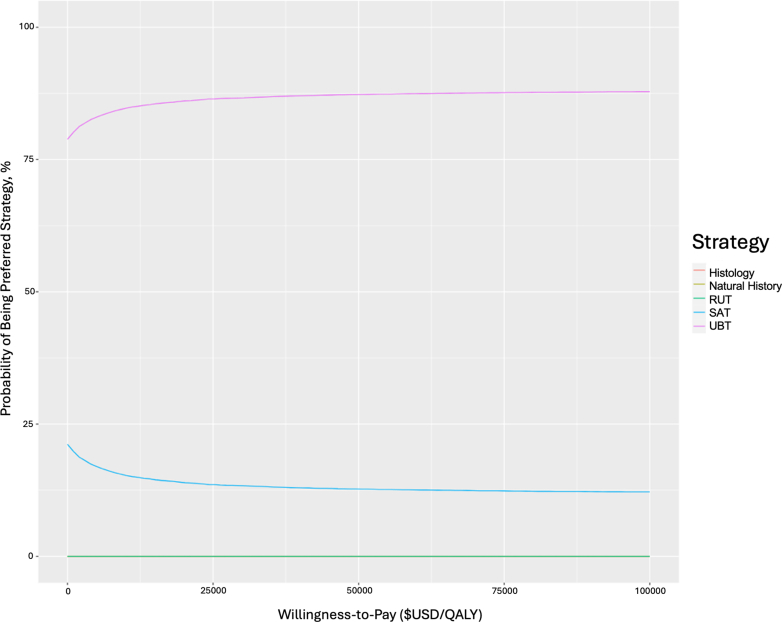
Probabilistic Sensitivity Analyses of Strategies at 17% H. pylori Positive Prevalence in Bleeding Peptic Ulcers.

In the base case version of our model at 17% H. pylori prevalence and a WTP threshold of $100,000/QALY, UBT was cost-effective across 87.8% of iterations. SAT was the cost-effective option in the remaining 12.2% of iterations. All other H. pylori testing strategies were cost-effective for 0% of iterations at any WTP threshold (Figure A2).

## Discussion

In this paper, we evaluate the cost-effectiveness of H. pylori testing at the time of endoscopy for bleeding PUD. Our analysis simultaneously compared 5 H. pylori testing strategies and no testing in an acute inpatient setting, which can often be difficult to obtain, to estimate and compare projected health outcomes to determine cost-effectiveness.^38^ Furthermore, PPIs, NSAIDs, and the presence of blood in the stomach can impact the performance of several H. pylori tests, making it difficult for clinicians to navigate which test to perform when treating a patient with PUD.^39^, ^40^, ^41^, ^42^, ^43^

Our analysis found UBT is the preferred H. pylori testing strategy in the inpatient setting from both an effectiveness and cost-effectiveness perspective. Our model results were robust, and UBT remained the cost-effective strategy across all 1-way sensitivity analyses and in 87.8% of all iterations in the probabilistic sensitivity analysis.

Histology, RUT, SAT, and serology resulted in higher costs and fewer QALYs than UBT. Although the cost of the UBT test alone is more expensive than other diagnostic tests, its efficacy in identifying H. pylori reduced recurrent hospitalizations, leading to an overall decrease in costs. In addition to being cost-effective, UBT may provide additional benefits not accounted for in this model, such as providing faster results than other options and patient preference.^44^ However, in the US, UBT may not be available for inpatient care; our analysis suggests that health-care systems can consider making this H. pylori testing modality available.

Our model expands upon previous work modeling the optimal H. pylori test in the inpatient setting. Firstly, we show that noninvasive testing strategies remain cost-effective across a vast range of H. pylori prevalence levels from 10% to 90%. Further, H. pylori prevalence has been steadily decreasing over the last decade. We estimate the base case H. pylori prevalence in the United States to be 17% while previous literature estimates this prevalence to be much higher (60%).^30^ Also, we include SAT as a possible strategy. As a result, we can recommend SAT as an alternative to UBT when it is not available. In our model, patients are only given PPI for the duration of their H. pylori treatment, which is better aligned with clinical recommendations.^45^ Lastly, we track patients over an extended period of time (35 years) after initial hospital admission.

An advantage of point-of-care H. pylori testing during index hospitalization is that deferred testing may ultimately not be completed if there is loss to follow up. Patients who test positive for H. pylori should be treated as soon as possible to reduce the future risk of additional peptic ulcer bleeding in the near term, and also to lower the risk of cancers, including gastric cancer, over a longer time horizon.^46^, ^47^, ^48^, ^49^ Although previous studies have shown that H. pylori eradication reduces the incidence of gastric cancer in asymptomatic patients,^50^^,^^51^ we did not include the downstream long-term benefits for gastric cancer prevention and other potential benefits. This is due to the limited data available and because such an analysis is beyond the scope of our study. Studies have also shown that H. pylori eradication prevents the future occurrence of PUD and dyspepsia, leading to increased quality of life.^52^

Our model demonstrates several key strengths. It reflects a common clinical scenario faced by many gastroenterologists. Further, our analysis addresses the concern that H. pylori testing in the context of an acute upper GI bleed, combined with PPI therapy, compromises test performance. The test performance characteristics in our model reflect those from a systematic review and meta-analysis in the setting of upper GI bleeding.^35^ Histology and RUT were the least sensitive and specific strategies while SAT and UBT were the most sensitive and specific strategies. Low performance of biopsy-based tests such as RUT and histology may be partially attributed to the uneven distribution of H. pylori throughout the stomach epithelium.^53^

### Limitations

The results of our analysis should be interpreted in the context of several limitations. As with any model, our results are limited to published data. Various factors may affect the accuracy of the diagnostic tests evaluated in our model. The performance metrics of these tests were calculated with the consideration that PPIs, NSAIDs, and the presence of blood in the stomach all impacted each test’s accuracy.^35^ However, there are many points of clinical variability not accounted for in the model, such as the duration of time between patient admission to endoscopy and the duration of PPI treatment.

While our cost-effectiveness analysis is based on the cost structures and health-care access models prevalent in the United States, the findings may vary in different settings. Differences in H. pylori prevalence, antibiotic resistance, costs, and health-care infrastructure could impact the cost-effectiveness of screening strategies. Adjusting the WTP threshold and H. pylori prevalence in our model could also yield varied results. Although beyond the scope of this US-based study, we plan to explore these factors in future analyses.

In this model, we do not consider outpatient follow-up testing for in-patient negatives, infections that are brought to attention and treated for reasons other than hospitalization, or complications other than bleeding (eg, perforation). Since bleeding comprises most complications, we do not expect these omissions to significantly impact our results.

## Conclusion

In this modeling analysis of patients hospitalized with bleeding peptic ulcers, noninvasive diagnostic tests for H. pylori proved to be more effective and cost-effective compared to invasive tests and no testing. Our analysis indicated that UBT was the most cost-effective strategy. Implementing noninvasive H. pylori testing at the point of care or during inpatient admission should be considered, as it poses minimal risk to patients and offers significant downstream clinical benefits.

## Supplementary Materials

Table A1

Table A2
