# Supplementary material for: Initial Diagnostic Strategies for Helicobacter Pylori in Patients With Bleeding Peptic Ulcers Undergoing Endoscopy: A Cost-Effectiveness Analysis
Source: Gastro Hep Adv. 2024 Dec 15;4(4):100602. doi: 10.1016/j.gastha.2024.100602 (PMC11849076; doi:10.1016/j.gastha.2024.100602)
Supplement: Table A1 [file mmc1.docx]

Supplemental Table 1: Model Input Parameters

| Parameter | Base-Case Estimate | Range Used in Sensitivity Analysis | Distribution for PSA | Sources |
| --- | --- | --- | --- | --- |
| Start Age | 65 | NA | NA | ^54^ |
| Percentage Male PUD Hospitalizations | 52.3% | 41.84–62.76%* | β | ^54^ |
| Prevalence of HP Positive Peptic Ulcers | 17% | NA | NA | ^30^ |
| **Probabilities** |  |  |  |  |
| Histology Sensitivity | 0.70 | 0.66–0.74 | β | ^35^ |
| Histology Specificity | 0.90 | 0.85–0.94 | β | ^35^ |
| RUT Sensitivity | 0.67 | 0.64–0.70 | β | ^35^ |
| RUT Specificity | 0.93 | 0.90–0.96 | β | ^35^ |
| Serology Sensitivity | 0.88 | 0.85–0.90 | β | ^35^ |
| Serology Specificity | 0.69 | 0.62-0.75 | β | ^35^ |
| Stool Antigen Sensitivity | 0.87 | 0.82–0.91 | β | ^35^ |
| Stool Antigen Specificity | 0.70 | 0.62–0.78 | β | ^35^ |
| UBT-13 Sensitivity | 0.93 | 0.90–0.95 | β | ^35^ |
| UBT-13 Specificity | 0.92 | 0.87–0.96 | β | ^35^ |
| HP Reinfection Rate | 0.0145 | 0.0116–0.0174* | β | ^55^ |
| HP Infection Rate | 0.0025 | 0.001–0.0063 | β | ^55^ |
| 1st Line Eradication Rate | 0.7 | 0.5–0.9 | β | ^56, 57^ |
| 2nd Line Eradication Rate | 0.81 | 0.62–1 | β | ^58^ |
| Risk of rebleeding, HP eradication, first year | 0.029 | 0.01–0.032 | β | ^59^ |
| Risk of rebleeding, HP eradication, subsequent years | 0.0015 | 0.0005–0.0036 | β | ^60^ |
| Risk of rebleeding, HP non-eradication, first year | 0.2 | 0.14–0.25 | β | ^59^ |
| Risk of rebleeding, HP non-eradication, subsequent years | 0.058 | 0.05–0.067 | β | ^59, 61, 62^ |
| Risk of rebleeding, HP negative, first year | 0.029 | 0.01–0.032 | β | ^59^ |
| Risk of rebleeding, HP negative, subsequent years | 0.0015 | 0.0005–0.0036 | β | ^60^ |
| Annual risk of bleeding, PUD | 0.025 | 0.02–0.03 | β | ^60^ |
| Lifetime risk of PUD, HP positive | 0.15 | 0.12–0.18* | β | ^60^ |
| 30-day mortality after peptic ulcer bleed | 0.086 | 0.058–0.114 | β | ^28^ |
| **Utilities** |  |  |  |  |
| HP Negative (healthy) | 1 |  |  |  |
| HP Positive | 0.9 | 0.8–1 | β | ^63^ |
| Peptic Ulcer Bleed | 0.5 | 0.4–0.6 | β | ^64^ |
| Death | 0 |  |  |  |
| **Costs** |  |  |  |  |
| Histology | $89.00 | $71.20–106.80*† | γ | ^65^ |
| RUT | $89.00 | $71.20–106.80*† | γ | ^65^ |
| Serology | $16.85 | $13.48–20.22* | γ | ^65^ |
| Stool Antigen | $14.38 | $11.50–17.27* | γ | ^65^ |
| UBT-13 | $105.47 | $84.38–126.56* | γ | ^65^ |
| 1st Line Eradication Therapy | $119.18 | $95.34–143.02* | γ | ^32^ |
| 2nd Line Eradication Therapy | $136.62 | $109.30–163.94* | γ | ^32^ |
| Hospitalization for PUD | $17,193.03 | $16,772.02–17,614.04 | γ | ^54^ |

**____________________________________________________________________________________________________________**

*±20% assumption; **Adjusted for inflation via Consumer Price Index; †Estimated as cost of EGD+Biopsy minus EGD alone
