# Supplementary material for: Initial Diagnostic Strategies for Helicobacter Pylori in Patients With Bleeding Peptic Ulcers Undergoing Endoscopy: A Cost-Effectiveness Analysis
Source: Gastro Hep Adv. 2024 Dec 15;4(4):100602. doi: 10.1016/j.gastha.2024.100602 (PMC11849076; doi:10.1016/j.gastha.2024.100602)
Supplement: Table A2 [file mmc2.docx]

Supplemental Table 2: Incremental Cost Effectiveness Ratios for all Six Strategies at Various H. pylori Positive Prevalence Levels in Bleeding Peptic Ulcers

| **Prevalence** |  |  |  |  |  |  |
| --- | --- | --- | --- | --- | --- | --- |
| 10% | **Strategy** | **Bleeds Avoided** | **Number Needed to Treat** | **Costs (Testing+ Drug Therapy + Hospitalization)** | **QALYs** | **ICER** |
|  | No Testing | Ref. | Ref. | $2098 | 16.734 | Ref. |
|  | RUT | 774 | 12.92 | $1119 | 16.873 | Dominated |
|  | Histology | 802 | 12.47 | $1084 | 16.879 | Dominated |
|  | SAT | 942 | 10.62 | $873 | 16.910 | Dominated |
|  | Serology | 949 | 10.54 | $865 | 16.911 | Dominated |
|  | UBT | 985 | 10.15 | $845 | 16.919 | -6756.48 |
|  |  |  |  |  |  |  |
| 30% | **Strategy** | **Bleeds Avoided** | **Number Needed to Treat** | **Costs (Testing+ Drug Therapy + Hospitalization)** | **QALYs** | **ICER** |
|  | No Testing | Ref. | Ref. | $5079 | 16.270 | Ref. |
|  | RUT | 2322 | 4.30 | $2132 | 16.715 | Dominated |
|  | Histology | 2407 | 4.15 | $2022 | 16.733 | Dominated |
|  | SAT | 2827 | 3.54 | $1430 | 16.825 | Dominated |
|  | Serology | 2849 | 3.51 | $1402 | 16.830 | Dominated |
|  | UBT | 2955 | 3.38 | $1292 | 16.853 | -6804.71 |
|  |  |  |  |  |  |  |
| 60% | **Strategy** | **Bleeds Avoided** | **Number Needed to Treat** | **Costs (Testing+ Drug Therapy + Hospitalization)** | **QALYs** | **ICER** |
|  | No Testing | Ref. | Ref. | $9550 | 15.642 | Ref. |
|  | RUT | 4644 | 2.15 | $3652 | 16.478 | Dominated |
|  | Histology | 4813 | 2.08 | $3429 | 16.514 | Dominated |
|  | SAT | 5655 | 1.77 | $2265 | 16.697 | Dominated |
|  | Serology | 5699 | 1.75 | $2207 | 16.707 | Dominated |
|  | UBT | 5910 | 1.69 | $1963 | 16.755 | -6816.77 |
|  |  |  |  |  |  |  |
| 90% | **Strategy** | **Bleeds Avoided** | **Number Needed to Treat** | **Costs (Testing+ Drug Therapy + Hospitalization)** | **QALYs** | **ICER** |
|  | No Testing | Ref. | Ref. | $14021 | 14.987 | Ref. |
|  | RUT | 6965 | 1.44 | $5172 | 16.242 | Dominated |
|  | Histology | 7220 | 1.39 | $4836 | 16.295 | Dominated |
|  | SAT | 8482 | 1.18 | $3100 | 16.570 | Dominated |
|  | Serology | 8548 | 1.17 | $3012 | 16.585 | Dominated |
|  | UBT | 8865 | 1.13 | $2633 | 16.656 | -6820.78 |
